# Current sleep interventions for shift workers: a mini review to shape a new preventative, multicomponent sleep management programme

**DOI:** 10.3389/frsle.2024.1343393

**Published:** 2024-02-08

**Authors:** Amber F. Tout, Nicole K. Y. Tang, Tracey L. Sletten, Carla T. Toro, Charlotte Kershaw, Caroline Meyer, Shantha M. W. Rajaratnam, Talar R. Moukhtarian

**Affiliations:** ^1^Division of Health Sciences, Warwick Medical School, University of Warwick, Coventry, United Kingdom; ^2^Department of Psychology, University of Warwick, Coventry, United Kingdom; ^3^Turner Institute for Brain and Mental Health, School of Psychological Sciences, Monash University, Melbourne, VIC, Australia

**Keywords:** sleep, intervention, shift work, mini review, prevention

## Abstract

**Introduction:**

Shift work can lead to sleep disturbances and insomnia during the sleeping period, as well as excessive sleepiness and fatigue during the waking period. While Cognitive Behavioral Therapy (CBT-i) is recommended as the first line of treatment for insomnia, key elements of CBT-i, such as maintaining a consistent sleep schedule, can be challenging for shift workers, highlighting the need for tailored sleep interventions. This mini review provides a narrative synthesis of non-pharmacological sleep interventions for shift workers and informs the development of a preventative, multicomponent sleep management programme.

**Method:**

An informal review was conducted in line with Phase 1 of the Framework for the Development and Evaluation of Complex Interventions.

**Results:**

A variety of strategies have been employed to help manage the impacts of shift work on sleep, including: CBT-i, adjusting shift schedules, controlled light exposure, sleep hygiene education, planned napping, caffeine consumption, and mind-body interventions (e.g., yogic relaxation).

**Discussion:**

Recommendations, limitations, and directions for future research are discussed; notably, the role of the family, the commute to and from the workplace, and the eating behaviors of employees appear to have been overlooked in current intervention efforts. Digital CBT-i platforms could help to provide an effective, scalable, and low-cost method of reducing insomnia in shift workers.

## 1 Introduction

Many organizations rely upon shift workers to maintain round-the-clock services and operations, with shift workers estimated to comprise 20% of the global workforce (Torquati et al., 2019; Booker et al., 2022). While shift schedules may vary greatly, individuals engaged in shift work are often required to work irregular hours and nights, resulting in a changeable sleep-wake pattern at odds with their body-clocks (circadian rhythms) and the world around them (Rajaratnam et al., 2013; Walker et al., 2020). The resulting “circadian misalignment” can lead to excessive sleepiness and fatigue during the waking period, as well as sleep disturbances and insomnia during the sleeping period (Chatterjee and Ambekar, 2017; James et al., 2017; Walker et al., 2020). When accompanied by significant distress and impairment, and not attributable to other underlying causes, these symptoms may be indicative of Shift Work Disorder (SWD), which is estimated to impact 26.5% of shift workers (Pallesen et al., 2021).

In addition to having a negative impact on sleep, circadian misalignment can also have detrimental consequences for physical health and psychological functioning, and has been associated with increased risk of cancer, cardiovascular diseases, and mood disorders (James et al., 2017; Torquati et al., 2019; Sletten et al., 2020; Walker et al., 2020). The effects of circadian misalignment can also be problematic for the workplace, leading to reduced levels of alertness and productivity, higher rates of sickness absence and staff turnover, and increased risk of accident and injury (Wong et al., 2011; Hui and Grandner, 2015; Ryu et al., 2017). Thus, as well as having a significant impact on the individual, the health and safety consequences associated with shift work also present a substantial socioeconomic burden (Culpepper, 2010).

When it comes to tackling insomnia in the general population, Cognitive Behavioral Therapy (CBT-i) is recommended as the first line of treatment (Edinger et al., 2021); however, elements of CBT-i – such as maintaining a consistent sleep schedule and sleep restriction – may not be possible for shift workers who work irregular hours and already experience sleep loss (Reynolds et al., 2022). Night workers who need to sleep during the day may also have difficulty accessing in-person therapies delivered during standard clinical hours. As a result, alternative strategies, such as adjusting shift schedules, controlled light exposure, and sleep hygiene education have been employed to help manage sleep problems in this population (Bragge et al., 2023).

Here, it should be noted that attempts to modify CBT-i for shift workers have been made more recently (Järnefelt et al., 2020), with digital platforms also providing 24/7 access options (Wickwire, 2019). Nevertheless, interventions that consider both the individual and the workplace may be more appropriate for this population, given that cognitive-behavioral factors and the organizational environment are likely to contribute to sleep problems in tandem (Papantoniou et al., 2017; Wong et al., 2019). Indeed, as well as focusing on individual-level factors, CBT-i targets those who are already engaged in shift work and/or already suffer from sleep problems as a result, highlighting the need to provide employees with the skills and knowledge to manage sleep from the outset of employment as part of a preventative approach (Wong et al., 2019).

Despite the existence of multiple strategies, however, efforts to combine and administer them as part of a preventative, multicomponent programme remain scarce, and research evidence for the effectiveness of organizational-level interventions is lacking (Redeker et al., 2019; Bragge et al., 2023). Given the widespread impacts of shift work and the associated costs to the individual, the workplace, and society, a preventative, multicomponent approach may therefore hold promise for improving the health and wellbeing of this population (Papantoniou et al., 2017; Wong et al., 2019).

Accordingly, this mini review aims to provide a concise overview of non-pharmacological interventions for shift workers to inform the development of a preventative, multicomponent sleep management programme in line with Phase 1 of the Framework for the Development and Evaluation of Complex Interventions (Skivington et al., 2021). To direct the review phase, co-authors with expertise in the areas of shift work and sleep pooled insights and relevant articles. Systematic reviews of the literature were explored to identify interventions; discussion papers, narrative reviews, and guidelines were consulted to determine current practice recommendations; and original intervention studies were examined for methodological details.

## 2 Narrative synthesis

**Figure 1:**
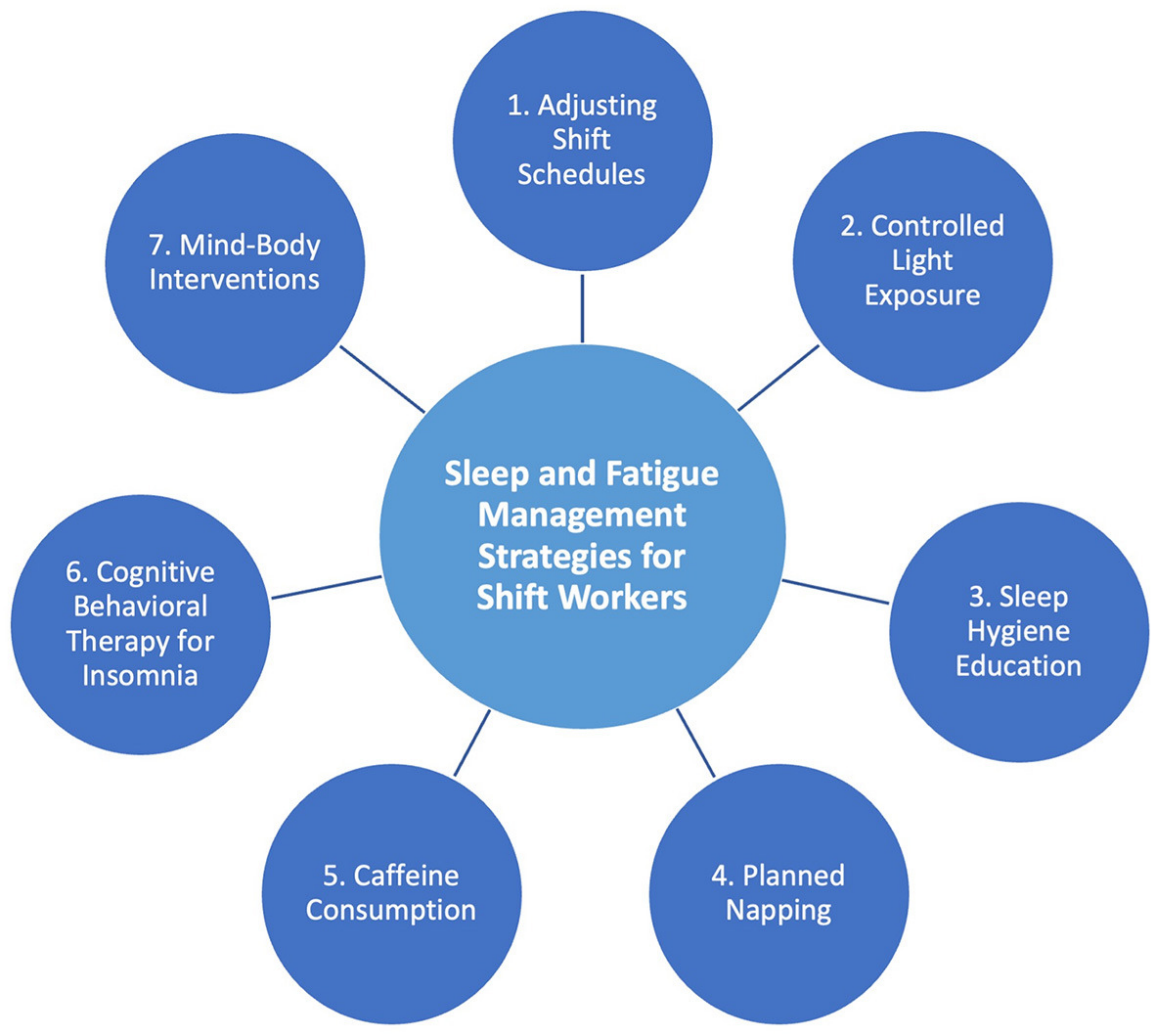
Current non-pharmacological sleep interventions for shift workers, most (1) to least (7) frequently implemented.

Systematic reviews have explored interventions for shift workers (e.g., Neil-Sztramko et al., 2014; Richter et al., 2016; Robbins et al., 2021; Bragge et al., 2023). The most-to-least frequently implemented strategies include: (1) Adjusting shift schedules; (2) Controlled light exposure; (3) Sleep hygiene education; (4) Planned napping; (5) Caffeine consumption;^1^ (6) CBT-i; and (7) Mind-body interventions (see Figure 1). A summary of the literature is provided in Supplementary Table 1.

### 2.1 Adjusting shift schedules

Adjusting shift schedules is one of the most frequently employed forms of intervention for shift workers. Here, it should be noted that interventions often modify multiple aspects of the shift in tandem (i.e., direction and speed of rotation, duration), making it difficult to determine the impact of specific adjustments.

#### 2.1.1 Rotation direction

Compared to backwards rotation (Night Shift → Evening Shift → Morning Shift), forwards rotation (Morning Shift → Evening Shift → Night Shift) has been shown to benefit sleep and alertness (Knauth and Hornberger, 2003; Bambra et al., 2008; Sallinen and Kecklund, 2010; Neil-Sztramko et al., 2014; Arlinghaus et al., 2019; Robbins et al., 2021; Bragge et al., 2023). Forward rotation accommodates the delay in timing of the circadian pacemaker and allows longer intervals between shifts, providing more time for recovery sleep and social activity (Garde et al., 2020).

#### 2.1.2 Rotation speed

Compared to slow rotation (7 Morning Shifts → 2 Days Off → 7 Evening Shifts → 2 Days Off → 7 Night Shifts → 2 Days Off), fast rotation (2 Morning Shifts → 2 Evening Shifts → 2 Night Shifts → 2 Days Off) has been shown to improve employee sleep (Knauth and Hornberger, 2003; Bambra et al., 2008; Sallinen and Kecklund, 2010). Fast rotation requires limited adjustment of circadian timing and enables swifter resynchronization to regular biological and social rhythms, while slow rotation makes an individual unavailable for social activity over a greater number of days (i.e., when working 7 consecutive nights; Arlinghaus et al., 2019).

#### 2.1.3 Self-rostering

Giving employees control over their work pattern can help to facilitate positive individual, social, and workplace outcomes by increasing autonomy and reducing stress (Knauth and Hornberger, 2003; Bambra et al., 2008; Garde et al., 2012, 2020; Arlinghaus et al., 2019). Indeed, intervention studies have shown that employees who specify their preferred shift start time, duration, and/or duties, exhibit improved mood, sleep, and recovery compared to those who do not, without compromising the total number of hours worked (Garde et al., 2020).

#### 2.1.4 Shift duration and timing

Research evidence for the optimal shift duration remains inconclusive; however, reducing shifts from ≥12 h to ≤ 10 h (or ≤ 9 h in the case of night shifts) has generally been shown to benefit sleep and reflects research-backed recommendations (Knauth and Hornberger, 2003; Sallinen and Kecklund, 2010; Arlinghaus et al., 2019; Wong et al., 2019; Garde et al., 2020; Bragge et al., 2023). Notably, reducing shift length has received a mixed reception in the healthcare sector, with fewer hours perceived as compressing workload and compromising education (Bragge et al., 2023). Nevertheless, 12 h shifts have not been shown to benefit staffing costs, productivity, performance, sickness absence, sleepiness, or wellbeing in this population (Dall'Ora et al., 2022).

To minimize circadian disruption, morning shifts should start as late as possible (i.e., 7 am vs. 6 am), while evening shifts should finish as early as possible (i.e., 10 pm vs. 11 pm; Knauth and Hornberger, 2003).

#### 2.1.5 Consecutive shifts and rest days

To improve employee outcomes and reduce the risk of fatigue-related accidents, consensus reviews recommend that individuals work a maximum of 5–7 consecutive shifts, and no more than 3 consecutive night shifts (Knauth and Hornberger, 2003; Garde et al., 2020). In instances where night work could pose greater risk (i.e., during pregnancy), 1 night shift per week is advised (Garde et al., 2020).

When it comes to rest periods between shifts, a minimum of 11hrs is recommended by the Working Time Society (Wong et al., 2019). To allow adequate time for recovery, days off should be scheduled to occur after night shifts, and single days off in-between workdays should be avoided; giving employees a 2-day off weekend equivalent per rotation can also protect work-life balance (Knauth and Hornberger, 2003; Wong et al., 2019).

#### 2.1.6 Chronotype

Individuals with morning chronotypes experience greater circadian misalignment on night shifts and may require additional on-shift interventions as a result, while individuals with later circadian phases exhibit better resilience and recovery (Sallinen and Kecklund, 2010; Juda et al., 2013). Accordingly, chronotype may be an important factor to consider when recruiting employees and allocating shifts; however, further research is needed to understand its effects on shift work tolerance and associations with age and sex (Ritonja et al., 2019).

### 2.2 Controlled light exposure

Light is essential to the regulation of the circadian system and has positive, non-visual effects on alertness, productivity, and sleep (Burgess et al., 2002). Accordingly, researchers have experimented with the timing, intensity, and color temperature of workplace lighting to help facilitate adaptation to night work or improve alertness and productivity (Robbins et al., 2021; Bragge et al., 2023). Indeed, the American Academy of Sleep Medicine recommended timed light exposure in the treatment of SWD (Morgenthaler et al., 2007).

Compared to standard lighting, blue-enriched light has been shown to improve sleep quality, alertness, concentration, and performance (Mills et al., 2007; Viola et al., 2008; Motamedzadeh et al., 2017; Sletten et al., 2021). Exposure to high intensity, blue-enriched light – particularly at the point of peak circadian sleepiness – can delay rhythms and facilitate adaptation to permanent or slow-rotating night shifts (Eastman and Martin, 1999; Horowitz et al., 2001; Burgess et al., 2002; Crowley et al., 2003). To prepare the body for the return to a “normal” sleep-wake pattern (i.e., on days off), the presentation of light can be timed to occur later each consecutive night shift (Eastman and Martin, 1999; Burgess et al., 2002). Here, it is important to note that employees on rapidly rotating shifts will not benefit from this gradual “circadian entrainment,” given the comparably slower nature of biological rhythms (Eastman and Martin, 1999).

To prevent morning light from advancing circadian rhythms and promoting wakefulness upon leaving the workplace, dark sunglasses can be worn on the commute home (Crowley et al., 2003); however, this may not be practical for those driving themselves, especially on dark mornings when the suppression of light could compromise safety. Once home, going straight to bed in a dark environment can improve sleep (Burgess et al., 2002). Avoiding light the morning before a night shift, where feasible, is also recommended (Morgenthaler et al., 2007).

### 2.3 Sleep hygiene education

SHE can cultivate awareness of healthy sleep practices; however, as is the case outside of the shift work population, evidence for its effectiveness as a standalone treatment for sleep problems in shift workers remains mixed (Robbins et al., 2021; Bragge et al., 2023), with many intervention studies reporting little to no effect on sleep and alertness (Arora et al., 2007; James et al., 2018; Pylkkönen et al., 2018; Booker et al., 2022).

SHE also tends to contradict accepted fatigue management strategies for shift workers (i.e., napping during the day and consuming caffeine on night shifts), while advice to maintain a consistent sleep-wake schedule fails to accommodate irregular working hours (Shriane et al., 2023). Accordingly, Shriane et al. (2023) recently utilized the Delphi method to develop a series of 18 “healthy sleep practices” for shift workers, wherein the unique challenges faced by this population were addressed; for instance, using napping as a tool to achieve 7 h−9 h of sleep per 24 h period. Determining the acceptability and effectiveness of these guidelines and/or incorporating them into multicomponent programmes present an opportunity for future research.

### 2.4 Planned napping

Napping before and/or during a night shift – particularly at the point of peak circadian sleepiness – can help to manage alertness and improve performance (Morgenthaler et al., 2007; Rajaratnam et al., 2013; Ruggiero and Redeker, 2014; Martin-Gill et al., 2018). Research suggests that a 20 min−30 min nap provides employees with the benefits of rest, whilst reducing the likelihood of entering deep sleep, which can lead to inertia upon awakening and compromise the subsequent sleep period (Ruggiero and Redeker, 2014; Martin-Gill et al., 2018). Nevertheless, employees may still need 15 min post-nap to regain cognitive faculties before resuming work (Ruggiero and Redeker, 2014; Martin-Gill et al., 2018).

### 2.5 Caffeine consumption

Like napping, the stimulating effects of caffeine have been shown to decrease sleepiness, reduce the risk of errors, and facilitate cognitive performance in the workplace (Ker et al., 1996; Temple et al., 2018). Regular, low dose caffeine consumption throughout the night shift counteracts performance deficits associated with extended wakefulness; however, consumption too near to the planned sleep period (i.e., < 6 h before) may disrupt sleep quality and duration (Wyatt et al., 2004; McHill et al., 2014).

### 2.6 Cognitive behavioral therapy for insomnia

Recommended as the first line of treatment for chronic insomnia in the general population, CBT-i has also been used to treat insomnia in shift workers. Findings from a recent meta-analysis revealed that CBT-i significantly reduced insomnia severity and improved sleep quality; however, values did not reach the threshold for clinical significance, and heterogeneity in intervention design, methodology, and delivery limited conclusions (Reynolds et al., 2022). High levels of attrition also suggest that current efforts may not sufficiently address the needs of this population (Reynolds et al., 2022).

Indeed, elements of CBT-i are challenging for shift workers, who may be unable to maintain consistent sleep schedules, rely upon naps to manage fatigue, and have restricted sleep durations (Järnefelt et al., 2012, 2020; Reynolds et al., 2022). Accordingly, researchers have attempted to modify CBT-i for this population by implementing a 4 h “anchor sleep” period instead of a consistent sleep schedule and allowing shift workers to have an extra hour in bed if their sleep duration is already restricted by work (Järnefelt et al., 2012, 2020). Other modifications involve adapting SHE to include advice on napping and light exposure, and emphasizing the use of relaxation techniques to reduce post-shift arousal (Järnefelt et al., 2012, 2020; Booker et al., 2022).

When it comes to accessing CBT-i, digital platforms could offer a relatively inexpensive and scalable option for employees and employers (Robbins et al., 2021). Encouragingly, 4–6 weeks of self-guided online CBT-i has been shown to improve insomnia symptoms in shift workers (Peter et al., 2019; Omeogu et al., 2020), although further research is needed to draw conclusions (Reynolds et al., 2022).

### 2.7 Mind-body interventions

Evidence for the effectiveness of mind-body interventions (e.g., mindfulness-based stress reduction, yogic relaxation, physical activity etc.,) remains limited within the shift work population (Robbins et al., 2021); however, a handful of studies have demonstrated that yogic relaxation and exercise can reduce stress and increase sleep quantity (Fang and Li, 2015; Raghul et al., 2018).

Given their effectiveness in other workplace settings (Pérez-Fuentes et al., 2020), mind-body interventions may be an additional element to consider incorporating into future multicomponent programmes (Robbins et al., 2021). Indeed, SHE and CBT-i encourage relaxation for reducing pre-sleep arousal, while mindfulness-based therapies for insomnia have proven to be effective in other working populations (Ong and Moore, 2020).

## 3 Discussion

This mini review highlights various strategies that have been employed to help manage the impacts of shift work on sleep; however, research evidence for the effectiveness of preventative, multicomponent programmes is currently lacking (Redeker et al., 2019; Bragge et al., 2023). A summary of the evidence pertaining to each intervention category is presented below; future directions and limitations are also considered.

Adjustments to shift schedules and changes to workplace lighting have been shown to mitigate the effects of shift work-related circadian misalignment; however, balancing the needs of employees against what is feasible for the organization may be complex (Wong et al., 2019). For instance, while reducing shift duration and enhancing workplace lighting might benefit employees, such organizational-level changes have substantial costs for employers (e.g., recruiting additional staff to make-up for lost hours). Thus, as well as emphasizing organizational benefits, government regulation of research-backed recommendations may be required to encourage implementation.

Given the health and safety consequences of shift work-related circadian misalignment, intervention programmes should aim to educate employers and equip employees with the skills needed to manage sleep from the outset of employment. Collective ownership of employee sleep could be achieved by delivering tailored forms of SHE as part of staff inductions and training. As employers are legally required to protect staff from health and safety risks, night workers could also be provided with personal protective equipment to facilitate daytime sleeping (e.g., eye-masks and earplugs).

In addition to the above, employers could consider facilitating nap opportunities and providing access to caffeine on nights shifts to help manage alertness. Advice on timing and dosage could also be incorporated into SHE, although negative attitudes toward “sleeping on the job” may present a barrier to the implementation of napping. While educating employers and being selective with terminology – i.e., “controlled rest” vs. “naps” – could help to overcome such challenges, providing suitable rest conditions may still pose difficulties for sectors with noisy and/or outdoor working environments. Indeed, it should be noted that the implementation of any intervention component must be tailored to job role, setting and circumstances.

As shift work increases the risk of clinical sleep problems, organizations should be prepared to support employees with the relevant resources. Digital CBT-i platforms could offer an inexpensive and scalable option for employers, while providing an external access option for employees who may have anxieties about revealing sleep problems in the workplace. Despite this, further research is needed to identify modifications that could improve the acceptability and effectiveness of digital CBT-i for shift workers (Reynolds et al., 2022). Similarly, the effectiveness of mind-body interventions remains unclear, despite benefits being found in other working populations (Pérez-Fuentes et al., 2020). While these approaches may help to address sleep problems associated with shift work-related circadian misalignment, it is also important to acknowledge their limited application to sleep disorders with other causal underpinnings (i.e., obstructive sleep apnea), which should be addressed via additional and/or alternative treatments (i.e., continuous positive airway pressure).

Lastly, it should be noted that personal demands also constrain sleep time and increase sleepiness (Knauth and Hornberger, 2003; Gurubhagavatula et al., 2021). Despite this, factors such as family life, the commute, and eating behaviors appear to have been overlooked in current intervention efforts. In light of this, working with shift workers and employers to co-develop preventative, multicomponent approaches represents an essential step for future research, and may help to identify further intervention opportunities (Wong et al., 2019; Reynolds et al., 2022).

## Author contributions

AT: Conceptualization, Investigation, Project administration, Visualization, Writing—original draft, Writing—review & editing. NT: Conceptualization, Funding acquisition, Writing—review & editing. TS: Conceptualization, Funding acquisition, Writing—review & editing. CT: Conceptualization, Funding acquisition, Writing—review & editing. CK: Conceptualization, Funding acquisition, Writing—review & editing. CM: Conceptualization, Funding acquisition, Writing—review & editing. SR: Conceptualization, Funding acquisition, Writing—review & editing. TM: Conceptualization, Funding acquisition, Supervision, Writing—original draft, Writing—review & editing.

## Footnotes

^1^As a widely available, prescription-free stimulant, caffeine is considered as a non-pharmacological intervention in this mini review.
