# Supplementary material for: Current sleep interventions for shift workers: a mini review to shape a new preventative, multicomponent sleep management programme
Source: Front Sleep. 2024 Feb 8;3:1343393. doi: 10.3389/frsle.2024.1343393 (PMC12713806; doi:10.3389/frsle.2024.1343393)
Supplement: Supplementary file 1 [file Table_1.docx]

| **Table 1.** *Literature Consulted to Inform Narrative Synthesis, Organized by Intervention Subtype.* | | | | | | |
| --- | --- | --- | --- | --- | --- | --- |
| 1. **Adjusting Shift Schedules** | | | | | | |
| **Author (Date)** | **Paper Type** | **Intervention Subtype** | **Population** | **Method** | **Key Outcome(s) and Results** | **Recommendation/Conclusion** |
| Arlinghaus et al., (2019) | Review Article – Working Time Society Consensus Statements for Shift Scheduling | Various Changes to Shift-Schedules | Various | Various | ***Work-Life Balance***  ***Social Wellbeing*** | Minimize long hours, on-call hours, evening & weekend work, and irregularity. Include flexibility and control over work hours. Fast, forward rotating shifts that allow some work-free evenings are preferable |
| Aust et al., (2023) | Systematic Overview of Systematic Reviews (957 primary studies from 52 reviews) for Organizational Level Changes | Various Organizational Level Changes (inc. changes to Shift-Scheduling) | Various | Various | ***Work-Life Balance, Health, Wellbeing***  4 strong quality reviews found good evidence that giving employees influence over their working time arrangements has positive effects on work outcomes, and some benefit to employee health and wellbeing. 4 reviews find moderate evidence that employee control over work tasks can improve employee health. | Give employees some control over their working time arrangements and the tasks they carry out when at work. |
| Bambra et al., (2008) | Systematic Review – Health Effects of Reorganizing Shift Work | Various Changes to Shift Schedules | Various | Various | ***Health, Sleep and Work-Life Balance***  Slow (i.e., 7 consecutive shifts) to fast (i.e., 3 consecutive shifts) and backward to forward rotation improves sleep/ health markers. Forward to backward rotation reduces sleep quality. Removing rotation and reducing night work improves outcomes. Self-scheduling benefits work-life balance/organizational effectiveness. | Switching from slow to fast rotation, changing from backward to forward rotation, and allowing some self-scheduling of shifts benefits outcomes. |
| Bragge et al., (2023) | Systematic Review of Sleep Health Interventions for Shift Workers | Various: Changes to Shift Schedules (N = 39) | Various | Various | ***Sleep and Mental Health***  Positive effects for forward/fast rotation and shortening shifts (e.g., 10hrs vs. 12hrs), but may increase work compression/decrease education time for healthcare workers. Negative effects of increasing shift duration. | Switching to a forward rotating and implementing shorter shifts/duty hour limits (10hrs vs. 12hrs) generally has a positive impact on outcomes. |
| Garde et al., (2012) | Experimental | Self-scheduling/Flexible Scheduling Intervention | 28 Workplaces (Healthcare Settings and Call Centers) | Baseline vs. 12-month follow-up. Intervention (A, B, C) vs. Reference.  A – choose start time and length, of shift; B and C – choose duties. | ***Working Hours, Recovery and Health***  Changes in working hours in A. Health improved in B. Less need for recovery in A and B. No changes in C.  No detrimental effects on need for recovery, sleep or health in A, B or C.  Did not compromise total number of hours worked or guidelines. | Self-rostering and considering employee preferences for start, length and duties improves work-life balance and social outcomes, without compromising total number of hours worked. |
| Garde et al., (2020) | Discussion Paper | Night Shift Scheduling Recommendations | Various | Various | ***Various*** | ≤3 consecutive night shifts. Night shift intervals ≥11hrs. ≤9 hours night shift duration. Pregnant women should not work more than 1 night shift per week. Forward rotation may be preferable. |
| Knauth and Hornberger (2003) | In-depth Review | Shift scheduling recommendations | Various | Various | - Avoid permanent night work. - ≤3-night shifts in succession. - At least 2 consecutive days off after last night shift. - Forward rotation pattern. - Max 5-7 consecutive days on. - Avoid single workdays in between days off. - At least 11hrs off between shifts. - Shifts of >8hrs only if necessary/if breaks are provided and adequate recovery time is allowed. - Not too early start morning shift (e.g., 6am vs. 5am). - Not too late finish evening shift (e.g., 10pm vs. 11pm). - End night shift as early as possible - Avoid work on weekends/provide 2-day off weekend equivalent if this is not possible. - Avoid irregularity and make flexibility/self-scheduling possible. | |
| Neil-Sztramko et al., (2014) | Systematic Review of Health-related Interventions for Night Shift Workers | Various: Shift scheduling Interventions (N = 15) | Various | Various | ***Sleep Quality and Quantity***  3/6 studies backward to forward rotation led to improvements in sleep.  3/6 studies – increasing duration from 8hr to 10hr or 12hr shifts led to no/negative changes in sleep. | Fast, forward rotating shifts tend to lead to more favorable sleep outcomes. Changes in shift duration and start-time are inconclusive and variable. |
| Richter et al., (2016) | Review of non-pharmacological interventions | Various: Shift scheduling Interventions | Various | Various | ***Sleep Duration, Jet Lag & Fatigue*** Morning types have more social jet-lag when working nights vs. evening types. More sleep in fast, rotating shifts | Chronotype should be taken into consideration i.e., assign evening types to night shifts.  Fast rotating = longer sleep. |
| Robbins et al., (2021) | Systematic Review of Workplace Employee Health Interventions | Various: Shift-scheduling Interventions (N = 5) | Various | Various | ***Sleep Duration*** Positive effects of moving to forward (or fast AND forward) rotation. Mixed effect of changing shift duration/breaks. | Forward (and fast) rotation may be better than backward. |
| Sallinen and Kecklund (2010) | Narrative Review | Shift Schedule Interventions | Various | Various | ***Various*** | Changing from slow, backward rotating shifts to fast, forward rotating shifts may be advantageous. Changes in shift length are inconclusive. |
| 1. **Controlled Light Exposure** | | | | | | |
| **Author (Date)** | **Paper Type** | **Intervention Subtype** | **Population** | **Method** | **Key Outcome(s) and Results** | **Recommendation/Conclusion** |
| American Association of Sleep Medicine (2007) | Guidelines for the Treatment of SWD | Lighting Guidelines | N/A | N/A | ***Sleep, Wellbeing & Circadian Phase Markers*** Studies show various forms of timed light exposure im­prove work performance, alertness, mood, and daytime sleep. Some also indicate that light shifts circadian rhythm phase markers (e.g., salivary melatonin). | Timed light exposure in the work environment and light restriction in the morning, when feasible, can help to decrease sleepiness and improve alertness during night shift work. |
| Bragge et al., (2023) | Systematic Review | Various Interventions: Lighting Exposure Studies (N = 12) | Various | Various | ***Sleep and Mental Health***  6 studies reported positive effects of light interventions on mood, sleep, wellbeing, and anxiety/depression. 1 study reported negative effect of daytime light blocking goggles on mood. 5 report no/variable effects on mood. | Evidence supports the use of lighting interventions to improve sleep and mood outcomes. |
| Burgess et al., (2002) | Clinical Review | Using bright light, dark and melatonin to promote circadian adaptation. | Various | Various | - Exposure to medium–high intensity light for 3–6hrs towards the beginning of a night shift (before the point of peak circadian sleepiness) can help employees adjust to consecutive night shifts. - Presenting light slightly later each night can push rhythms back in sync with natural day/night cycle in prep for switching back to daily life - Sunglasses on route back from night shift to avoid daylight advancing rhythms. - Sleep when you get in from a night shift (e.g., 8am to 3pm) and keep a regular wake/sleep schedule when on shifts. - Exposure to bright light on days off can help realign cycle with normal life. | |
| Crowley et al., (2003) | Intervention | Light – assessing combinations of bright light, sunglasses, and melatonin | 67 “young participants” (simulated night shift work, not shift workers) | Baseline week vs. intervention week (6 conditions with different combinations of lighting intervention). | ***Circadian Re-entrainment***  Circadian re-entrainment (adaptation to night shift work) indicated by dim light melatonin onset and Tmin. | A combination of bright light in the workplace, sunglasses on the commute home and a dark bedroom environment facilitated circadian entrainment. Those with early phases (i.e., ‘morning’ types) benefited the most from intervention. Addition of melatonin made no difference. |
| Eastman and Martin (1999) | Review | Using light and dark to adapt to night work | Various | Various | - Exposure to bright light (for at least 3hrs) before the point of peak sleepiness can help to delay rhythms/suppress melatonin. Bright light after this point may risk advancing rhythms. - Bright light should be moved later each day to facilitate adaptation back to daily life when night shifts have ended. - Medium intensity light may be as effective as high intensity light. - Light exposure on way home can override effects, so wear dark goggles on route home and sleep straight away to facilitate entrainment to the night shift. - Re-entrainment is best achieved by bright light during night shift/goggles on the way home combination. | |
| Horowitz et al., (2001) | Intervention | Light – bright vs. room light exposure + fixed vs. free sleep schedules | 54 participants (not shift workers – simulated night shifts) | 4 Conditions: Bright light + Fixed Sleep; Bright light + Free Sleep; Room light + Fixed sleep; Room light + Free Sleep | ***Circadian Re-entrainment & Alertness*** Bright light + fixed sleep had positive, additive effects and led to circadian re-entrainment (adaptation to night shift) indicated by dim light melatonin onset, subjective alertness + actigraphy. Those who adopted a fixed sleep schedule in the free sleep condition exhibited more entrainment/improvements. | Use of both bright light exposure and fixed sleep-wake schedule on night shifts facilitate best circadian adaptation to night shift work. |
| Mills et al., (2007) | Intervention | Light – high correlated color temperature fluorescent lighting vs. control | 69 office workers (day shifts) | Higher color temperature (17000K about 40 lux brighter) vs. Baseline color temperature (2900K) | ***Fatigue Symptoms & Sleepiness***  Self-reported concentration was the only outcome to sig. improve vs. control group at study end. Direction of changes were same in control arm. | Led to a wide range of trending improvements, but not significantly more than the control group. |
| Motamedzadeh et al., (2017) | Intervention | Light – blue enriched white light | 30 control room staff (night workers) | Baseline lighting vs. 1-week of 17000K (blue-enriched white light) vs. 1-week of 6500K | ***Sleepiness, Cognitive Performance & Salivary Melatonin***  Sleepiness and melatonin levels sig. declined in 17000K/blue-enriched white light condition vs. baseline and 6500K condition. Errors also decreased, and attention improved. | The use of blue-enriched white light may help to improve alertness and performance during night shifts. |
| Neil-Sztramko et al., (2014) | Systematic Review | Various Interventions: Light Interventions (N = 12) | Various | Various | ***Sleep Quality and Quantity***  8/10 studies had some significant effect on at least 1 sleep outcomes and/or provided evidence of a phase shift (indicated by melatonin, body temp and/or cortisol response). 5/6 studies that included sunglasses/blue-light blocking elements led to improvement in some sleep/shift outcome. | A combination of timed bright light and light blocking goggles promote adaptation to shift work. While many studies occurred in artificial work environments, consistency across studies suggests that multi-pronged interventions to control light exposure may be more effective than bright light or light-blocking goggles alone. |
| Richter et al., (2016) | Narrative Review | Various Reviews/Interventions: Light Interventions | Various | Various | ***Various*** | Companies should consider light exposure timing and intensity. Light restriction in the morning may also help to delay before a night shift. |
| Robbins et al., (2022) | Systematic Review | Various Lighting Exposure Studies (N = 5) | Various | Various | ***Sleep Duration***  4/5 light exposure interventions led to increases in sleep duration. | Light exposure is a well-documented method of shifting circadian phase and helps in the adaptation to night shifts. |
| Sletten et al., (2017) | Intervention | Light – 17000K (blue enriched light) vs. 4000K standard light | 71 night shift workers with a variety of occupations | 2 nights shifts + 1 simulated night shift with either 17000K blue-enriched light or 4000K standard light | ***Circadian Phase, Objective & Subjective Sleepiness, Performance*** 17000K light did not sig. improve performance or reduce sleepiness vs. standard light. However, subjective sleepiness was reduced relative to circadian phase in 17000K condition. | Blue-enriched light may help to improve subjective sleepiness ratings in night workers. The timing of light may be an important factor to consider (i.e., at the point of peak sleepiness). Further research is needed in the selection of light properties to maximize benefits. |
| Sletten et al., (2021) | Intervention | Light | 28 shift workers from a chemical plant | 2 light conditions on 2 consecutive nights: Standard light (4000K, 43 lux) vs. higher intensity blue-enriched light (17000K, 106 lux) | ***Subjective Sleepiness, Mood, Performance***  Sleepiness was sig. attenuated and performance was better in the 17000K condition vs. the 4000K condition. Mood was not affected by condition. | Exposure to blue-enriched light led to improvements in alertness and performance. Light could be used as a countermeasure for impaired alertness during night shifts. |
| Viola et al., (2008) | Intervention | Light | 94 white collar workers across 2 floors | Cross-over design – 4wks 17000K (blue enriched light), followed by 4000K (standard light). | ***Alertness, Performance, Mood, Fatigue, Concentration, Sleepiness***  Blue-light improved subjective measures of alertness, mood, performance, fatigue, irritability, concentration and eye discomfort. Daytime sleepiness and sleep quality also improved. Expectation effects may have affected some outcomes. | Blue-enriched lighting in the workplace environment can have positive effects on employee alertness, mood and fatigue/sleepiness. |
| 1. **Sleep Hygiene Education** | | | | | | |
| **Author (Date)** | **Paper Type** | **Intervention Subtype** | **Population** | **Method** | **Key Outcome(s) and Results** | **Recommendations/Conclusion** |
| Arora et al., (2007) | Intervention | SHE | 58 Medical Interns | 60-90min SHE/fatigue management programme | ***Sleep***  No beneficial effect on sleep. | Regardless of SHE, medical interns continue to be sleep deprived as a result of duty-hour regulations. |
| Booker et al., (2022) | Intervention | Individualized sleep and shift work education programme | 149 nurses identified as high risk of SWD | 8-week shift work individualized management programme vs. active control (low GI diet programme) | ***Sick leave, Sleep and Wellbeing***  No sig. reduction in sick leave vs. active control. Improvements in sleep hygiene, insomnia and depression occurred in both groups. Anxiety and functional outcomes of sleep improved for intervention only. | An individualized sleep and shift work education programme did not improve sleep outcomes vs. an active control that were provided with education about low GI diet. |
| Bragge et al., (2023) | Systematic Review | Various: SHE (N = 3) | Various | Various – education/coaching encompasses the science of sleep, sleep disorders and sleep strategies | ***Sleep and Mental Health.*** 2/3 had positive effects on quality of life, burnout and psychological distress. 3/3 had positive effects on sleep outcomes. | Despite significantly improving sleep and mental health outcomes, the current evidence base for SHE is too small to draw firm conclusions. |
| Holzinger et al., (2019) | Intervention | Sleep Coaching | 30 shift workers with various occupations from a railway company | Pre (baseline) vs. post (6 months) 2-day sleep coaching seminar, including dream work, and relaxation techniques | ***Sleep and Burnout***  Sleep coaching significantly improved total PSQI score, subjective sleep quality and daytime sleepiness. No improvement was observed for sleep duration or burnout. | Sleep coaching may be effective at improving sleep quality in shift workers future research with larger sample sizes is needed. |
| James et al., (2018) | Intervention | SHE | 61 police officers | 3-4 hours of fatigue management training in groups over 4 weeks. Training includes science of sleep, sleep disorders, fatigue countermeasures and SHE | ***Sleep, Insomnia and Wellbeing***  Fatigue management training led to increased satisfaction with sleep fewer symptoms of insomnia and fewer headaches no effect on PSQI quality of life or psychological distress | SHE holds promise for improving sleep health and wellness of employees. The creation of restorative rest areas for strategic napping and guiding supervisors to better support employees who are chronically fatigued via increased monitoring and awareness is needed. |
| Nakada et al., (2018) | Intervention | SHE | 71 office-based employees | Pre vs. Post – Control vs. SHE programme in the workplace – 3x50min lectures on basic sleep knowledge, sleep disorders and interventions for good sleep, confirmation of skill. | ***Sleep and Insomnia***  Weekday sleep duration increased for the intervention group six months later. There was no effect on sleep efficiency or latency, sleepiness, health, insomnia or quality of life between or within groups. | SHE may help to improve weekday sleep. Interventions must involve the whole organisation/employer to facilitate programmes. |
| Neil-Sztramko et al., (2014) | Systematic Review | Various: SHE (N = 1) | Various | Educational programme about strategies to enhance adaptation to shift work for emergency department attending physicians | ***Sleep Quality and Quantity***  Led to an increase in REM asleep | Good lifestyle habits may not arise spontaneously among shift workers as a result of shift schedule changes. Interventions targeted at improving lifestyle/sleep behaviors may therefore be necessary |
| Pylkkönen et al., (2018) | Intervention | SHE | 53 truck drivers | A single 3½ hour lecture on the basics of sleep alertness management techniques followed by workshop. Personalized advice also given based upon schedules. Access to a sleep consultant for a further two months | ***Alertness and Sleep***  No effect on alertness sleepiness amount of prior sleep or the use of efficient management techniques during shifts | Failed to provide support non-recurrent alertness management training. Results suggest that driver education alone is not a sufficient measure to improve sleep and alleviate driver sleepiness. |
| Richter et al., (2016) | Review | SHE | Various | Various | ***Various*** | SHE should be provided to employees as part of a multicomponent intervention. Involving the family may help to facilitate behavioral change. |
| Robbins et al., (2021) | Systematic review | Various: SHE (N = 5) | Various | Various interventions with elements of SHE | ***Sleep Duration***  2/5 interventions found positive effects of sleep hygiene education on sleep outcomes | Emerging consensus that SHE is insufficient to improve sleep on its own and should be used in combination with CBT-i techniques, particularly in the presence of insomnia symptoms. |
| Shriane et al., (2023) | Guidelines – “Healthy Sleep Practices” for Shift Workers | SHE | 55 sleep/ shift work experts completed 3 rounds of review to generate consensus guidelines. | Delphi Method was used to develop 18 SHE guidelines for shift workers. | - Guideline 1: Prioritize your sleep. - Guideline 2: Aim for 7-9 hours of sleep per 24hrs. - Guideline 3: Develop a sleep schedule. - Guideline 4: Develop a bedtime routine. - Guideline 5: Plan your transition to days off. - Guideline 6: Use napping as a helpful tool. - Guideline 7: Consider sleep inertia. - Guideline 8: Create a comfortable sleep environment. - Guideline 9: Use your bed for sleep and intimacy only. - Guideline 10: Consider light exposure. - Guideline 11: Consider caffeine intake. - Guideline 12: Consider nicotine consumption. - Guideline 13: Consider alcohol intake. - Guideline 14: Be mindful of medication. - Guideline 15: Consider food intake. - Guidelines 16: Consider fluid intake. - Guidelines 17: Engage in regular exercise. - Guideline 18: Develop strategies for sleep problems. | |
| 1. **Planned Napping** | | | | | | |
| **Author (Date)** | **Paper Type** | **Intervention Subtype** | **Population** | **Method** | **Key Outcome(s) and Results** | **Recommendations/Conclusion** |
| AASM (2007) | Guidelines for the treatment of SWD | Napping Guidelines | N/A | N/A | ***Alertness, Performance & Accidents***  Studies using laboratory simulations and field investigations of shift work have shown that pre-shift napping and on-shift napping may help to improve alertness and reaction time, and decrease accidents during night shifts without affecting post-shift daytime sleep. | Planned napping before or during the night shift has been shown to performance and alertness-related outcomes on night shifts. |
| Bragge et al., (2023) | Various: Napping Interventions (N = 5) | Napping | Various | Various | ***Sleep and Mental Health***  Mixed results – 3 studies found negative or no effects of naps on mood, 2 studies found naps led to more vigor and decreased anxiety. Sleep outcomes improved in 4/5 studies. | Modest sample sizes and wide variation in nap timing (i.e., 10min. to 120min.) restricts conclusions. Napping may be easier to implement in healthcare settings vs. construction sectors where there is no place to nap and/or it can be seen as lazy. |
| Bonnefond et al., (2001) | Intervention | Napping | 12 male shift workers – electric plant | Allowed to have a 1hr rest period (in bedrooms) between 11.30pm and 3.30am. Followed-up over a year. | ***Sleep and Fatigue***  Naps did not reduce the main sleep period after the shift. Satisfaction with sleep quality progressively improved. Shift workers adapted and did not want to go back to no naps. Greater ease staying awake and less fatigue in night hours. | Naps can be implemented to reduce fatigue in the middle hours of a night shift and improve satisfaction with sleep quality. |
| Centofanti et al., (2016) | Intervention | Napping | 31 Healthy Participants | 30min nap vs. 10min nap vs. no nap at 4am on 3 consecutive simulated night shifts | ***Performance, Sleepiness, Mood***  Naps did not have an effect on performance. 30min nap led to sig. improvement in subjective sleepiness vs. 10min and no-nap condition. 10min nap worsened mood vs. no-nap and 30min nap. No change in objective sleepiness. | A 30min. nap at 4am helped to improve subjective sleepiness. A 10min. nap may lead to worsened mood vs. no nap due to sleep inertia. |
| Chang et al., (2015) | Intervention | Napping | 63 nurses | 30min nap between 2-3am on a night shift vs. no nap | ***Performance & Anxiety***  No effect of nap vs. no nap | Napping did not lead to benefits for performance or anxiety in nurses |
| Della Rocco et al., (2000) | Intervention | Napping | 65 air traffic controllers | Long nap (2hr) vs. short nap (45min) vs. no nap on night shift | ***Performance, Sleepiness, Mood***  2hr and 45min naps protected performance and reduced sleepiness during a midnight shift. Long nap resulted in more consistent findings. No effect on mood. Nap during peak circadian sleepiness may be better. | Naps during night shifts can help to protect wake-related performance degradation and reduce sleepiness – particularly at the point of peak circadian sleepiness. |
| Martin-Gill et al., (2017) | Systematic Review and Meta-analysis | Napping: (N = 13) | Various | Various | ***Performance, Fatigue, Sleep***  Naps benefitted performance in 8/11 studies and fatigue in 5/11 studies. Naps had no impact on sleep in 7/9 studies and had an unfavorable impact in 1. | Studies provide for the performance-related benefits of napping on night shifts; however, benefits to sleep remain unclear and heterogeneity in nap length are likely to influence results. |
| Purnell et al., (2002) | Intervention | Napping | 24 male aircraft engineers | Given 20min nap opportunity between 1am and 3am on 2-night shifts during experimental week vs. no naps on baseline week | ***Performance, Sleep and Fatigue***  Nap on first night shift led to sig. improvement in performance. No sig. effect on sleep or fatigue. | Short naps may help to counteract wake-related performance deficits on night shifts. Effects on sleep and fatigue remain inconclusive. |
| Rajaratnam et al., (2011) | Review | Various: Includes napping | Various | Various | - 30min–2hr nap the evening before a night shift can help to supplement main sleep episode. - Naps of 20-30min during the night may help to maintain wakefulness and alleviate excessive sleepiness on the shift (particularly in high-risk professions e.g., driving). - Nap opportunity must allow individuals adequate time to shake off sleep inertia – for a nap period of up to 60min, there should be a 15min interval before returning to work. | |
| Richter et al., (2016) | Systematic Review | Various: Napping (N = 5) | Various | Various | ***Sleep Duration and Jet Lag***  Cites reviews and papers mentioned here that recommend 20-30min naps on-shift and 30min-2hr naps pre-night-shift. | Naps are recommended, but efficacy depends on timing and duration as well as the stage of sleep and the circadian phase (could cause inertia/impair vigilance). |
| Rosekind et al., (1995) | Review | Alertness Management: Strategic naps | Various | Various | - Prophylactic naps – naps taken prior to a period of sustained wakefulness (i.e., night shifts) help to maintain alertness and performance. - Operational naps – naps taken on shift can also help to improve alertness/mood, sustain wakefulness, and alleviate sleepiness. - Inertia must be considered – appears to dissipate after 10-15min – entering slow wave sleep contributes to inertia, so limiting nap time could help (i.e., under 40min). - Naps could potentially delay the later sleep period. | |
| Ruggiero and Redeker (2014) | Narrative Systematic Review | Naps (N = 13) | Various | Various | ***Reaction Time & Subjective Sleepiness***  30, 40, 50min naps between midnight and 1am led to faster reaction time, fewer lapses and less subjective sleepiness at the end of a shift (N = 3).  60 or 120min nap did not improve reaction time (N = 1). 20, 30, 40, 60 or 120min naps between 2-3am improved outcomes (N = 7)  30 or 50min naps at 4am improved sleepiness, but inertia was greater. | 20–40min naps between 2am and 3am show feasibility in the workplace and improve outcomes, but sleep inertia must be considered. |
| Smith-Coggins et al., (2006) | Intervention | Naps | 49 physicians and nurses | 40min nap at 3am vs. no nap | ***Performance, Alertness, Driving***  Napping improved performance, alertness and mood at the end of the shift vs. no nap. Memory temporarily worsened immediately after the nap (inertia). Nap group did not do better on a simulated drive home. | Napping may help to improve performance and subjective measures of alertness and mood. |
| Takeyama et al., (2002) | Intervention | Naps | 60 male students (simulated shifts) | Naps between 2am-4am vs. no nap | ***Sleepiness & Anxiety***  Standard increases in sleepiness and anxiety associated with prolonged wakefulness were attenuated for morning types who napped. Not as big an influence on evening types. | Napping may be an effective strategy to reduce wake-related performance degradation and anxiety for those who identify as a morning type. |
| 1. **Caffeine Consumption** | | | | | | |
| **Author (Date)** | **Paper Type** | **Intervention Subtype** | **Population** | **Method** | **Key Outcome(s) and Results** | **Recommendations/Conclusion** |
| Ker et al., (2010) | Systematic Review and Meta-analysis | Various Caffeine Intervention Studies (N = 13) | Various (individuals with jetlag or SWD) | Various | ***Performance, Sleep and Alertness***  Across studies, caffeine helped to reduce the number of errors vs. placebo across concept formation and reasoning, memory, orientation and attention, perception (no effect on verbal/language skills). No sig. difference between caffeine and other interventions on these outcomes, but potentially better alertness outcomes vs. napping. 2 studies found caffeine led to subsequent sleep disruption, 2 did not. | Caffeine can help to improve performance and reduce fatigue, but there is potential disruption to subsequent sleep periods. Studies to date have not assessed the impact of caffeine on injury outcomes. |
| McHill et al., (2014) | Intervention | Caffeine | 30 healthy adults (simulated night shift work) | Individuals were given 2.9mg/kg of caffeine during the circadian trough (~5 hours before a daytime recovery sleep period). | ***Body Temperature, Alertness, Sleep*** Caffeine sig. increased body temperature, alertness, and clear-headedness on shift, but disturbed daytime recovery sleep. Higher body temp and wider skin temp gradient prior to sleep was associated with a longer latency to sleep, and disturbed recovery sleep (i.e., increased wakefulness after sleep onset, increased stage 1 sleep, decreased sleep efficiency, and decreased slow wave sleep). | Caffeine can be used to increase alertness on a night shift, but associated increases in body temperature may result in disruption to the subsequent sleep period. |
| Temple et al., (2018) | Systematic Review and Meta-analysis | Various Caffeine Intervention Studies (N = 8) | Various | Various | ***Performance, Sleep and Fatigue***  Caffeine improved performance on psychomotor vigilance test.  2/2 studies indicated a favorable impact on personnel safety. 2/2 studies indicated an unfavorable impact on sleep quality and quantity (reduction in duration and perceived quality vs. placebo).  3/4 studies reported reductions in fatigue. 1 report no impact. | Caffeine can help to reduce fatigue and maintain performance/alertness, but possibly at the cost of sleep. |
| Wyatt et al., (2004) | Intervention | Caffeine | 16 healthy men (simulated night shift work) | 0.3mg/kg of caffeine per hour during a 28.57hr wake episodes vs. placebo. | ***Performance and Sleep***  Rising caffeine levels attenuated wake-dependent performance deterioration, particularly at the circadian performance nadir (drop). Caffeine enhanced extended wakefulness and reduced risk of sleep, but at the expense of increasing subjective sleepiness. | Caffeine may help to prevent wake-related performance deterioration, but at the risk of inhibiting later sleep and increasing subjective sleepiness. |
| 1. **Cognitive Behavioral Therapy for Insomnia (CBT-i)** | | | | | | |
| **Author (Date)** | **Paper Type** | **Intervention Subtype** | **Population** | **Method** | **Key Outcome(s) and Results** | **Recommendations/Conclusion** |
| Bragge et al., (2023) | Systematic Review | Various: CBT-i (N = 5) | Various | Various | ***Sleep and Mental Health***  All 5 studies found significant effects of CBT-i on mental health and/or sleep outcomes. | Despite significant effects, variability in sample size, population, CBT-i design and content makes it difficult to draw conclusions of effectiveness. |
| Jang et al., (2020) | Intervention | CBT-i (+ image reversal therapy for nightmares) | 39 firefighters | Pre vs. Post CBT-i + image reversal therapy for nightmares | ***Sleep, Insomnia and Wellbeing***  Improvements in sleep efficiency and reductions in sleep onset latency, number of awakenings and time in bed. Reduced depression, PTSD & insomnia. | Tailored therapy for insomnia and nightmares may help to alleviate sleep problems in this population, but further research is needed. |
| Järnefelt et al., (2012) | Intervention | CBT-i | 19 shift workers (media) with non-organic insomnia | Pre vs. Post modified CBT-i for shift workers – group sessions and individual sessions delivered in person by trained nurses. Modified sleep restriction, anchor period and sleep hygiene advice. | ***Sleep and Wellbeing***  Improvements in mental health-related quality of life, self-reported and actigraphy-assessed sleep onset latency, self-reported sleep efficiency and quality. Reductions in perceived insomnia severity, sleep-related dysfunctional cognitions, psychiatric and somatic symptoms. | CBT-i can be implemented among shift workers with insomnia; however small sample sizes and non-randomized design limit conclusions. |
| Järnefelt et al., (2020) | Intervention | CBT-i | 83 shift workers with insomnia (various) | Partially randomized – group CBT-i vs. self-help CBT-i vs. sleep hygiene education (control) | ***Sleep and Wellbeing***  Insomnia, sleep-related dysfunctional beliefs, burnout, rest, recovery and total sleep time (actigraphy assessed) improved across all conditions. Those with SWD improved more. Mood improved only for the group CBT-i condition. | Improvements occurred in all conditions – mood improvements were the main added value for group CBT-i vs. self-help CBT-i and/or SHE control |
| Lee et al., (2014) | Intervention | Home-based “sleep enhancement training system” based on principles of CBT-i | 21 nurses | Prospective longitudinal design – within subjects – 4 week active control followed by 4 week intervention. CBT-i based principles. | ***Sleep and Wellbeing***  Significant improvement in self-reported (but not actigraphic) sleep quality over time. Improved wellbeing (fewer depressive symptoms and reduced anxiety). | Demonstrated feasibility of home-based therapy and the ability to improve self-reported sleep and mental health outcomes, but high attrition and small sample limits conclusions. |
| Omeogu et al., (2020) | Intervention | CBT-i | 13 nurses with symptoms of insomnia | Pre vs. Post self-guided CBT-i via Coach app for 6 weeks | ***Insomnia severity***  Significantly decreased between baseline and the 3 and 6 week follow-up (no sig. difference in score between 3 and 6 weeks). | Self-guided CBT-i led to reductions in insomnia severity in nurses |
| Peter et al., (2019) | Intervention | CBT-i | 21 shift workers with symptoms of insomnia | Pre vs. Post, 4-week online CBT-i delivered via email with access to a professional via phone or email vs. 6 face-to-face outpatient CBT-i sessions | ***Sleep, Insomnia and Wellbeing***  Improvements in sleep efficiency for both conditions (no sig. differences between them). Insomnia and wellbeing scores improved over the course of the online programme. No change in sleepiness for either condition. Reductions in depression and improvements in wellbeing and sleep for face-to-face CBT-i. | Online CBT-i may be as effective as improving sleep efficiency as face-to-face treatment. High drop-out rates, small sample and lack of randomization make conclusions difficult to draw. |
| Reynolds et al., (2022) | Systematic Review and Meta-analysis | CBT-i Interventions (N = 9) | Various | Various | ***Insomnia Severity and Sleep Quality***  Meta-analysis of 6 studies revealed CBT-i led to significant reduction in insomnia symptoms and improvements in global PSQI scores (but not minimally clinical importance difference). | The limited number of studies, small sample sizes, high levels of attrition and heterogeneity mean there is insufficient evidence at present to recommend CBT-i as a treatment for insomnia in shift workers. Efforts to co-develop modified forms are needed. |
| Richter et al., (2016) | Review | Various: CBT-i (N = 1) | Various: Media workers | CBT-i (see Järnefelt et al., 2012) | ***Sleep and Wellbeing.*** | CBT-i may be useful for improving long-term sleep quality in shift workers. |
| Robbins et al., (2021) | Systematic Review | Various: CBT-i (N = 1) | Various: nurses | CBT-i (see Lee et al., 2014) | ***Sleep and Wellbeing*** | CBT-i may be effective, but in-person delivery requires significant time and resources for employees and employers. Low-cost, scalable digital platforms may help to address this. |
| 1. **Mind-Body Interventions** | | | | | | |
| **Author (Date)** | **Paper Type** | **Intervention Subtype** | **Population** | **Method** | **Key Outcome(s) and Results** | **Recommendations/Conclusion** |
| Bragge et al., (2023) | Systematic Review | Various: Yoga (N = 1) | Various: Healthcare professionals | Yogic Relaxation (see Raghul et al., 2018) | ***Various***  Reductions in stress | Despite benefits to stress levels, there is not enough evidence from a single study to draw wider conclusions. |
| Fang and Li (2015) | Intervention | Yoga | 120 Healthcare assistants | Yoga intervention | ***Sleep***  Improvement in self-reported sleep duration based on PSQI component. | Yoga may help to improve sleep in shift workers |
| Härma (1988) | Intervention | Physical Exercise | 75 nurses | Physical training programme (2-6x a week). | ***Body composition, sleep,***  Improved subjective sleep duration, body composition and oxygen volume. | Promoting exercise may be beneficial for shift workers. |
| Morgan et al., (2011) | Intervention | Weight loss | 110 Plant workers | Group-based weight loss programme: 1-on-1 information session, study website, booklet and financial incentive | ***Body composition, diet and exercise***  Benefits observed for body composition, blood pressure, physical activity and diet. No impact on fizzy drink consumption/alcohol risk score. | Interventions targeting weight loss via healthy lifestyle practices may be beneficial for shift workers. |
| Neil-Sztramko et al., (2014) | Review | Various:  Physical activity (N = 1)  Weight loss (N = 1) | Various | Physical activity  (see Härma et al., 1988)  Group-based weight loss programme (see Morgan et al., 2011) | ***Sleep duration/social jet-lag***  Physical activity improved sleep duration but had a variable effect on sleep quality. No effect on circadian phase as measured by body temperature. Weight loss intervention reduced body mass/blood pressure and increased physical activity/fruit intake. | Interventions specifically targets at improving lifestyle behaviors may be necessary for encouraging healthy habits in shift workers. |
| Raghul et al., (2018) | Intervention | Yogic relaxation | 35 healthcare professionals | Pre vs. post 30min of yogic relaxation | ***Stress***  Stress was significantly reduced | Yoga may help to reduce stress levels |
| Robbins et al., (2021) | Systematic Review | Various: Yoga (N = 1) and Exercise (N = 1) | Various | Yogic relaxation (see Fang and Li, 2015).  Exercise (see Härma 1988) | ***Sleep duration***  Yoga and exercise interventions both increased sleep duration | Shift workers may benefit from widely available, low-cost digital applications. |

*Note.* CBT-i = Cognitive Behavioral Therapy for Insomnia; GI = Glycaemic Index; PSQI = Pittsburugh Sleep Quality Index; PTSD = Post Traumatic Stress Disorder; REM = Rapid Eye Movement sleep; SHE = Sleep Hygiene Education; sig. = significant effect; SWD = Shift Work Disorder.

**References:**

Aust, B., Moller, J.L., Nordentoft, M., Frydendall, K.B., Bengtsen, E., Jensen, A.B., Garde, A.H., Kompier, M.A.J., Semmer, N., Rugulies, R. and Jaspers, S.O., 2023. How effective are organizational-level interventions in improving the psychosocial work environment, health, and retention of workers? A systematic overview of systematic reviews. DOI: [10.5271/sjweh.4097](https://doi.org/10.5271/sjweh.4097)

Bonnefond, A., Muzet, A., Winter-Dill, A.S., Bailloeuil, C., Bitouze, F. and Bonneau, A., 2001. Innovative working schedule: introducing one short nap during the night shift. *Ergonomics*, *44*(10), pp.937-945. DOI: [10.1080/00140130110061138](https://doi.org/10.1080/00140130110061138)

Centofanti, S.A., Hilditch, C.J., Dorrian, J. and Banks, S., 2016. The impact of short night-time naps on performance, sleepiness and mood during a simulated night shift. *Chronobiology international*, *33*(6), pp.706-715. DOI: <https://doi.org/10.3109/07420528.2016.1167722>

Chang, Y.S., Wu, Y.H., Lu, M.R., Hsu, C.Y., Liu, C.K. and Hsu, C., 2015. Did a brief nap break have positive benefits on information processing among nurses working on the first 8-h night shift?. *Applied Ergonomics*, *48*, pp.104-108. DOI: [10.1016/j.apergo.2014.11.005](https://doi.org/10.1016/j.apergo.2014.11.005)

Della Rocco, P.S., Comperatore, C., Caldwell, L. and Cruz, C.E., 2000. *The effects of napping on night shift performance* (No. DOT/FAA/AM-00/10). Civil Aeromedical Institute.

Jang, E.H., Hong, Y., Kim, Y., Lee, S., Ahn, Y., Jeong, K.S., Jang, T.W., Lim, H., Jung, E., Shift Work Disorder Study Group and Chung, S., 2020. The development of a sleep intervention for firefighters: the FIT-IN (Firefighter’s therapy for insomnia and nightmares) Study. *International journal of environmental research and public health*, *17*(23), p.8738. DOI: [10.3390/ijerph17238738](https://doi.org/10.3390%2Fijerph17238738)

Lee, K.A., Gay, C.L. and Alsten, C.R., 2014. Home-based behavioral sleep training for shift workers: a pilot study. *Behavioral sleep medicine*, *12*(6), pp.455-468. DOI: [10.1080/15402002.2013.825840](https://doi.org/10.1080/15402002.2013.825840)

Purnell, M.T., Feyer, A.M. and Herbison, G.P., 2002. The impact of a nap opportunity during the night shift on the performance and alertness of 12‐h shift workers. *Journal of sleep research*, *11*(3), pp.219-227. DOI: 1[0.1046/j.1365-2869.2002.00309.x](https://doi.org/10.1046/j.1365-2869.2002.00309.x)

Rosekind, M.R., Smith, R.M., Miller, D.L., Co, E.L., Gregory, K.B., Webbon, L.L., Gander, P.H. and Lebacqz, J.V., 1995. Alertness management: strategic naps in operational settings. *Journal of sleep research*, *4*, pp.62-66. DOI: [10.1111/j.1365-2869.1995.tb00229.x](https://doi.org/10.1111/j.1365-2869.1995.tb00229.x)

Sletten, T.L., Ftouni, S., Nicholas, C.L., Magee, M., Grunstein, R.R., Ferguson, S., Kennaway, D.J., O’Brien, D., Lockley, S.W. and Rajaratnam, S.M., 2017. Randomised controlled trial of the efficacy of a blue-enriched light intervention to improve alertness and performance in night shift workers. *Occupational and environmental medicine*, *74*(11), pp.792-801. DOI: [10.1136/oemed-2016-103818](https://doi.org/10.1136/oemed-2016-103818)

Smith-Coggins, R., Howard, S.K., Mac, D.T., Wang, C., Kwan, S., Rosekind, M.R., Sowb, Y., Balise, R., Levis, J. and Gaba, D.M., 2006. Improving alertness and performance in emergency department physicians and nurses: the use of planned naps. *Annals of emergency medicine*, *48*(5), pp.596-604. DOI: [10.1016/j.annemergmed.2006.02.005](https://doi.org/10.1016/j.annemergmed.2006.02.005)

Takeyama, Hidemaro, Tomohide Kubo, and Toru Itani. "The nighttime nap strategies for improving night shift work in workplace." *Industrial health* 43, no. 1 (2005): 24-29. DOI: <https://doi.org/10.2486/indhealth.43.24>
